# Probing protein aggregation at buried interfaces: distinguishing between adsorbed protein monomers, dimers, and a monomer–dimer mixture *in situ*†

**DOI:** 10.1039/d1sc04300e

**Published:** 2021-12-21

**Authors:** Tieyi Lu, Wen Guo, Prathamesh M. Datar, Yue Xin, E. Neil G. Marsh, Zhan Chen

**Affiliations:** Department of Chemistry, University of Michigan Ann Arbor Michigan 48109 USA zhanc@umich.edu

## Abstract

Protein adsorption on surfaces greatly impacts many applications such as biomedical materials, anti-biofouling coatings, bio-separation membranes, biosensors, antibody protein drugs *etc.* For example, protein drug adsorption on the widely used lubricant silicone oil surface may induce protein aggregation and thus affect the protein drug efficacy. It is therefore important to investigate the molecular behavior of proteins at the silicone oil/solution interface. Such an interfacial study is challenging because the targeted interface is buried. By using sum frequency generation vibrational spectroscopy (SFG) with Hamiltonian local mode approximation method analysis, we studied protein adsorption at the silicone oil/protein solution interface *in situ* in real time, using bovine serum albumin (BSA) as a model. The results showed that the interface was mainly covered by BSA dimers. The deduced BSA dimer orientation on the silicone oil surface from the SFG study can be explained by the surface distribution of certain amino acids. To confirm the BSA dimer adsorption, we treated adsorbed BSA dimer molecules with dithiothreitol (DTT) to dissociate these dimers. SFG studies on adsorbed BSA after the DTT treatment indicated that the silicone oil surface is covered by BSA dimers and BSA monomers in an approximate 6 : 4 ratio. That is to say, about 25% of the adsorbed BSA dimers were converted to monomers after the DTT treatment. Extensive research has been reported in the literature to determine adsorbed protein dimer formation using *ex situ* experiments, *e.g.*, by washing off the adsorbed proteins from the surface then analyzing the washed-off proteins, which may induce substantial errors in the washing process. Dimerization is a crucial initial step for protein aggregation. This research developed a new methodology to investigate protein aggregation at a solid/liquid (or liquid/liquid) interface *in situ* in real time using BSA dimer as an example, which will greatly impact many research fields and applications involving interfacial biological molecules.

## Abstract 2

SFG spectra analyzed by the developed Hamiltonian method show that adsorbed BSA molecules on silicone oil are dimers. On treatment with dithiothreitol, some BSA dimers dissociate, resulting in 60% dimer and 40% monomer on the silicone oil surface.

## Graphical abstract

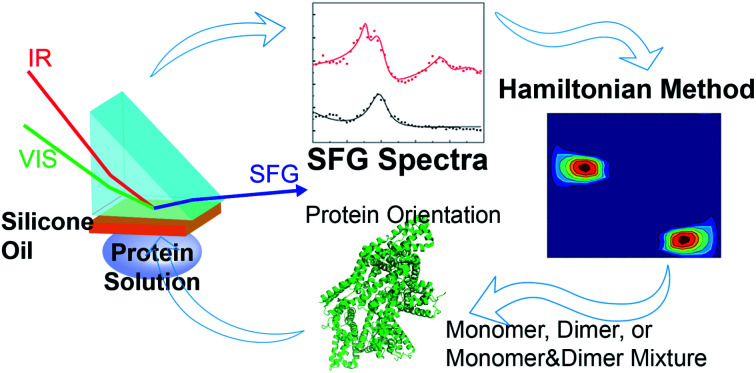

## Introduction

Protein molecular behavior on surfaces or at interfaces determines protein properties and function, and greatly impacts many applications and research fields, especially those involving interfacial proteins, such as biosensors, anti-fouling coatings, biomedical implants, and membranes used for bio-separation, and protein antibody drugs.^1–11^ In order to rationally design surfaces/interfaces to optimize interfacial protein properties and functions through controlling protein interfacial behavior, it is important to develop powerful tools to investigate the molecular structure and molecular behavior of proteins at various interfaces. Many analytical techniques have been developed for characterizing protein structures and behavior, such as X-ray diffraction, nuclear magnetic resonance (NMR), attenuated total reflection-FTIR (ATR-FTIR), cryo-electron microscopy (cryo-EM), quartz crystal microbalance (QCM), *etc.*, leading to important knowledge on protein molecules.^12–14^ However, such techniques have some disadvantages in investigating protein molecules at buried solid/liquid or liquid/liquid interfaces *in situ* in real time. They either lack surface/interface sensitivity or require high vacuum to operate and are thus unable to be used for *in situ* studies, or cannot provide detailed structural information at the molecular level.

Sum frequency generation vibrational spectroscopy (SFG) is a unique and powerful second-order nonlinear vibrational spectroscopic tool,^15–36^ which can detect amide I signals from interfacial protein molecules, providing molecular level information regarding the protein structure.^18,19,31,37,38^ According to the selection rule of a second-order nonlinear optical process, SFG is a highly surface/interface-sensitive method with a sub-monolayer specificity.^15–39^ It allows probing protein behavior at buried solid/liquid and liquid/liquid interfaces *in situ* directly.^18,19,31,39,40^ SFG can monitor interfacial adsorption of proteins in real time and probe detailed structural information of interfacial proteins (such as protein orientation).^18,19,31,37–41^ In previous studies by our group, we have reported the successful detection and orientation analysis of many proteins at different interfaces, including cell membrane associated proteins,^42^ surface chemically immobilized enzymes,^43^ and physically adsorbed proteins.^37,40^

In recent years, antibody drugs have been developed into important cures for many diseases, including cancers, autoimmune diseases, and chronic inflammatory diseases.^44^ Since generally an antibody only binds to a corresponding antigen, antibody drugs are highly efficient and selective, which makes the antibody treatment a powerful method to treat tumors and other diseases that require targeted agents. More and more antibody drugs have become commercially available. During antibody drug storage and administration, these protein drug molecules have opportunities to contact various surfaces. Silicone oil is commonly used as a lubricant in the pharmaceutical industry, such as a coating material for the wall of a syringe for protein antibody drug storage and injection. It has been shown that silicone oil can induce protein adsorption and possible aggregation, and thus potentially reduce the efficacy of protein drugs.^45–47^ It is thus important to study protein behavior on the silicone oil surface *in situ* at the molecular level, which has been rarely reported due to the lack of appropriate methods which can be used to study interfacial proteins at the silicone oil/solution interface *in situ* at the molecular level.

In this study, we applied SFG to study interfacial protein behavior at the silicone oil/protein solution interface *in situ* in real time at the molecular level, using bovine serum albumin (BSA) as a model. BSA has been widely used as a model protein for studying protein adsorption and interfacial protein behavior in various systems.^48–51^ It has also been widely used as a passivation coating for different surfaces.^52^ Despite the large number of publications reporting BSA interfacial adsorption, *in situ* studies on the detailed BSA interfacial structure at the molecular level are still rare. Previous research has shown that adsorbed BSA can adopt a dimer form – for example, it was reported that BSA molecules can form dimers in aged solutions through di-sulfide bonds, which causes protein aggregation and fouling on membranes.^53^ In these studies, HPLC was frequently used to study the BSA dimer formation, but such an experimental approach cannot be used to probe adsorbed protein molecules on the surface *in situ* to elucidate the protein interfacial behavior directly at the molecular level.^54–56^

SFG spectra were directly measured from the adsorbed BSA at the silicone oil/protein solution interface. These SFG spectra were analyzed with a Hamiltonian local mode approximation method.^57–60^ We have shown in our previous publication that such a Hamiltonian data analysis approach can be used along with SFG results to deduce interfacial protein orientation and conformation.^60^ Using the Hamiltonian method, we can calculate the SFG spectra of adsorbed BSA as a function of orientation. The calculated BSA orientation dependent SFG spectra of the BSA monomer and dimer are markedly different.

The comparison between the measured SFG spectra and calculated SFG spectra leads to the identification of whether the BSA monomer or dimer is present at the interface and the determination of adsorbed BSA monomer or dimer orientation. We also showed that this method can be used to study BSA monomer–dimer mixtures at the buried silicone oil/protein solution interface. Dimerization is a crucial initial step for protein aggregation.^61–63^ Our research demonstrates that with the help of the Hamiltonian data analysis method, SFG can be used to study protein aggregation *in situ*. Previously we used SFG to study the adsorption behavior of fusion protein and antibody onto a silicone oil surface, but no detailed structural information was reported.^64,65^ The methodology developed in this study is generally applicable and can be used to study the aggregation of many different proteins including fusion and antibody protein drugs at interfaces. It is worth mentioning that protein aggregation plays significant roles in many biological and biomedical process.^66–70^

## Results and discussion

### Sum frequency generation vibrational spectroscopy (SFG)

Details of SFG instrumentation and SFG theories have been extensively published^15–43,71–77^ and will not be reported here. Our SFG experiments were conducted using an EKSPLA PL 2250 SFG system (Vilnius, Lithuanian). To generate a sum frequency signal, a 532 nm visible beam and a frequency tunable IR beam (wavelength ranges from 2.3 to 10 μm) were overlapped spatially and temporally on the sample surface/interface. The sample geometry used in this study to collect SFG spectra from the silicone oil/solution interface is shown in the ESI (Fig. S1).† Time-dependent SFG signals were first collected by tracking the signal intensity of the characteristic peak from α-helices in BSA after the silicone oil surface was placed in contact with the BSA solution (with a 1 s time step). After the adsorption reached an equilibrium (evidenced by a constant SFG signal intensity), static SFG spectra were collected in ssp (s-polarized SFG signal, s-polarized visible beam, and p-polarized input IR beam) and ppp polarization combinations. The SFG spectra collected from each sample interface are very reproducible (see the ESI, Section S1†). All the SFG spectra presented here are averaged by three scans. The resolution of the SFG spectrometer is about 5 cm^−1^.

### Hamiltonian data analysis and protein orientation determination method

We applied the Hamiltonian data analysis method to calculate the SFG polarized (*e.g.*, ssp and ppp) spectra of BSA as a function of BSA orientation. A schematic to show the Hamiltonian method has been published previously^60^ and a revised schematic is included in the ESI (Section S2).† For BSA with a certain orientation, the Hamiltonian method started with a data matrix with diagonal elements as the peak centers of the uncoupled amide I signal of each amino acid (*e.g.*, 1645 cm^−1^) and off-diagonal elements with couplings between each pair of the amino acids. Such a matrix can be diagonalized to find the normal modes of the BSA amide I signals and their related peak centers. The IR transition dipole moment and Raman polarizability components of each normal mode can then be obtained, from which the SFG hyperpolarizability elements of each normal model can be calculated. Using the peak centers and the hyperpolarizability elements of each normal mode, along with an estimated peak width for each normal mode vibrational peak, SFG polarized spectra can be calculated. Then BSA can be rotated, and orientation dependent SFG spectra can be calculated. In this study, we applied the Hamiltonian method to calculate BSA orientation dependent SFG ssp and ppp spectra using the crystal structure of BSA dimer (PDB:4f5s) or monomer (chain A in PDB:4f5s) as the input file. The SFG spectra of BSA were calculated with the tilt angle range from 0 to 180°, and the twist angle range from 0 to 360°. The calculation angle step size for the tilt or twist angle is 10°. The reference orientations (0, 0 – with both tilt and twist angles of zero) for the BSA dimer and monomer are defined by using the BSA dimer and monomer orientations shown in the PDB (PDB:4f5s and chain A in 4f5s). Such reference orientations are shown in the ESI (Fig. S7).†

The calculated orientation dependent SFG spectra were then compared to the SFG resonant spectra extracted from the experimental data after deconvoluting the non-resonant contribution. The best match between the calculated and fitted spectra provides the most likely protein orientation. In this study, to find the most probable BSA orientation, the calculated SFG spectra and the fitted resonant spectra from experimental data are normalized in intensity (with the highest signal in the spectrum) to compare the ssp or ppp spectrum. We calculated the sum of the square of the difference between each data point in a fitted experimental ssp (or ppp) spectrum and a calculated ssp (or ppp) spectrum and used this summed square error to generate a heat map to describe the matching quality between the experimental data and the calculated spectra as a function of protein orientation. This heat map compares the spectral features of the ssp (or ppp) spectrum between the calculated and experimentally collected spectra. In addition to the above comparison standards shown by the two heat maps for the ssp and ppp spectral features, an additional standard of the signal intensity ratio of the ssp and ppp spectra was used to match the experimental data and the calculated spectra. Here we used the difference between the ssp and ppp peak intensity ratio at 1645 cm^−1^ (the highest intensity) of the calculated and fitted experimental spectra. The square of such a difference obtained as a function of protein orientation generates a heat map to address the ssp and ppp intensity ratio difference between the calculated and the experimental data. The combined heat map generated by the above three heat maps (one for intensity ratio, and two for ssp and ppp spectral features respectively) exhibits the overall difference between the calculated and experimentally measured spectra as a function of protein orientation. This heat map can be converted to a final heat map to quantify the matching score (using the biggest difference to subtract the difference for each protein orientation). The most likely protein orientation can be identified from the final matching score heat map. More details of the above matching process can be found in the ESI.†

The most likely BSA orientation can be determined based on an overall heat map which combines the heat maps for the ssp spectrum, ppp spectrum, and ssp/ppp signal strength ratio.

### SFG experiment result

**Fig. 1:**
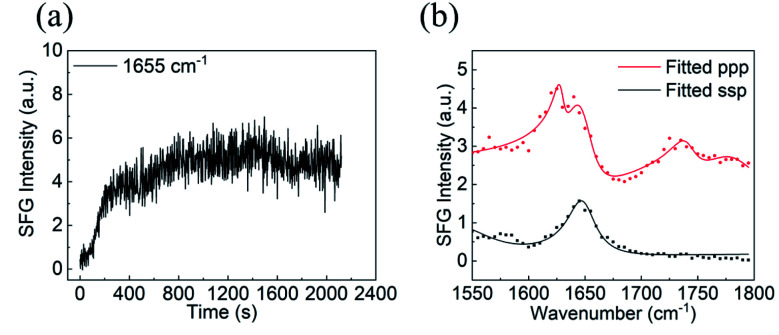
(a) Time-dependent SFG ssp signal intensity observed at ∼1650 cm^−1^ during the BSA adsorption process onto silicone oil, (b) SFG ssp and ppp spectra of adsorbed BSA collected from the silicone oil/BSA solution interface. The spectra are offset.

No SFG signal could be detected between 1500 and 1800 cm^−1^ from silicone oil in air and in water. After the BSA solution was placed in contact with silicone oil, SFG signal intensity at ∼1650 cm^−1^ increased at the silicone oil/BSA solution interface (Fig. 1a), showing that BSA molecules adsorbed onto the silicone oil surface. The SFG signal intensity increased very fast in the first several minutes, then slowly increased and more or less reached a plateau after 20 minutes (Fig. 1a), indicating the BSA adsorption reached equilibrium. SFG ssp and ppp spectra were then collected from the silicone oil/BSA solution interface (Fig. 1b). The amide I signals from adsorbed BSA on silicone oil can be clearly seen. The peak at around 1645 cm^−1^ in both ssp and ppp spectra is attributed to the alpha-helical structure in BSA. We also noticed that there is a second peak around 1630 cm^−1^ in the ppp spectrum, which is contributed by other structures in the protein.

**Fig. 2:**
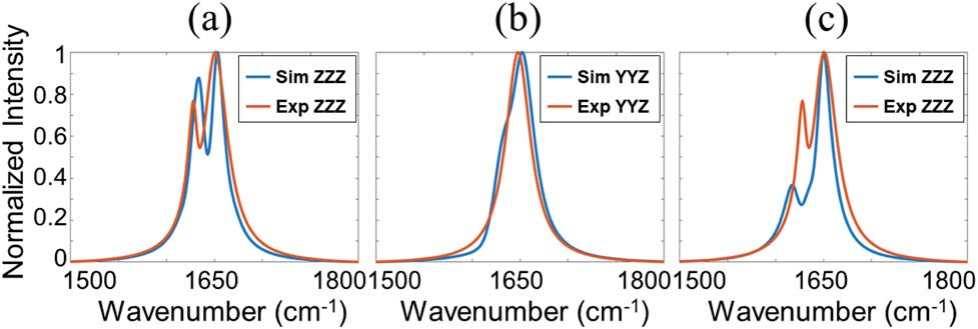
Comparison between simulated and experimental SFG spectra. (a) Red: reconstructed resonant SFG ppp spectrum from the experiment data. Blue: calculated ppp SFG spectrum based on the BSA dimer. (b) Red: reconstructed resonant SFG ssp spectrum from the experiment data. Blue: calculated ssp SFG spectrum based on the BSA dimer. (c) Red: reconstructed resonant SFG ppp spectrum from the experiment data. Blue: calculated ppp SFG spectrum based on the BSA monomer.

Since the collected SFG spectra contain a non-resonant contribution, we cannot compare the experimental data to the calculated spectra using the Hamiltonian method directly. It is necessary to extract the resonant spectral contribution from the experimental data. We fitted the SFG spectra using the standard SFG spectral fitting method^77^ and reconstructed the resonant SFG spectra using the fitted parameters (Fig. 2a and b, red spectra), which were used to compare to the calculated spectra.

### Orientation and structure analysis by the Hamiltonian method

Before we present the detailed SFG data analysis results of BSA on silicone oil, it is worth mentioning that BSA–silicone oil interactions can be complicated, leading to the existence of BSA monomers as well as oligomers (*e.g.*, dimers, trimers, tetramers, *etc.*) at the silicone oil interface. In the bulk environment, it has been shown that the BSA dimer is the most probable oligomer form,^78^ which is also shown by our PAGE experiments (see the ESI†). Here we hypothesize that the dominating BSA forms on silicone oil are monomer and/or dimer forms. Our data analysis shown below demonstrates that the calculated SFG spectra based on the structures of the BSA monomer and/or BSA dimer could match well with the experimental data, showing that the above hypothesis is valid. However, we could not completely rule out the formation of other oligomers, which will not be considered in this research.

**Fig. 3:**
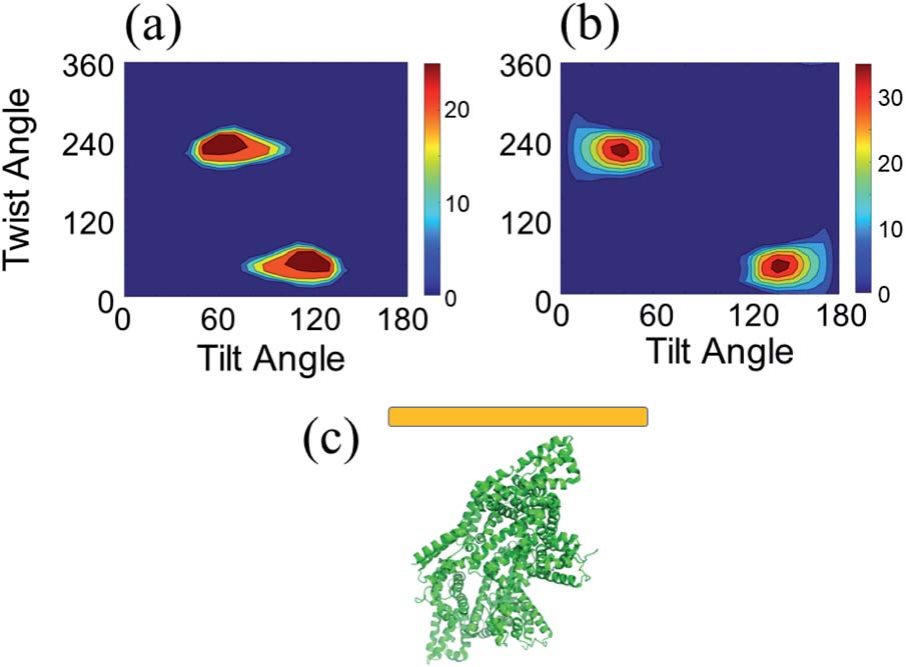
(a) Heat map showing the matching quality between the reconstructed resonant SFG spectra and calculated orientation dependent SFG spectra based on the BSA monomer. (b) Heat map showing the matching quality between the reconstructed resonant SFG spectra and calculated orientation dependent SFG spectra based on the BSA dimer. (c) Most likely orientation of BSA dimer on silicone oil. Each heat map shows two most likely orientations, with different absolute orientations (up and down). Since BSA dimer has two identical monomers, it is not necessary to differentiate the two orientations.

We calculated the SFG ssp and ppp spectra of BSA as a function of BSA orientation using the Hamiltonian method based on the BSA monomer crystal structure and BSA dimer crystal structure. The calculated SFG spectra were compared to the reconstructed resonant SFG spectra from the experimental data using the method presented in the method section above to create heat maps. Fig. 3a and b display the final score heat maps generated from the BSA monomer and dimer respectively. Clearly the highest matching scores in the heat map generated based on the BSA dimer are much higher compared to those in the heat map generated based on the BSA monomer, indicating that adsorbed BSA does not have monomer as the dominating form, which is different from the bulk solution case (see PAGE results in the ESI†). Based on our hypothesis stated above, it is likely that BSA mainly adopts the dimer form on the silicone oil surface. Fig. 3b shows that the BSA dimer orientation with a tilt angle of 40° and a twist angle of 220° has the highest matching score, which is the most likely orientation of the BSA dimer on silicone oil. This orientation is plotted in Fig. 3c. The calculated SFG ssp and ppp spectra from the most likely BSA dimer orientation (tilt angle of 40° and twist angle of 220°) are plotted in Fig. 2a and b, and are very similar to the reconstructed resonant SFG ssp and ppp spectra from the experimental data. For comparison, the SFG ppp spectrum calculated from the most likely orientation in the heat map deduced based on the BSA monomer is plotted in Fig. 2c, which is more different from the experimentally generated resonant ppp spectrum compared to that based on the BSA dimer (Fig. 2a).

**Fig. 4:**
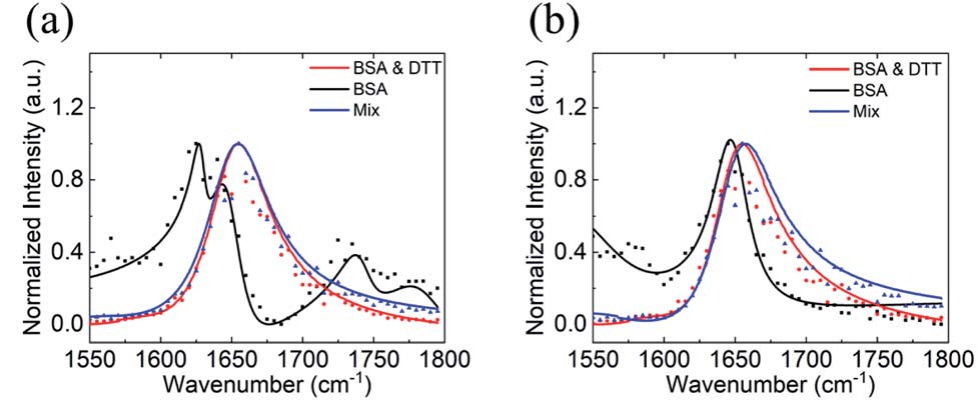
SFG ppp (a) and ssp (b) spectra of BSA with DTT treatment. The SFG spectra collected from BSA before the DTT treatment, BSA after the DTT treatment, and BSA pre-mixed with the DTT are displayed in black, red, and blue color, respectively.

A BSA dimer can be formed by the di-sulfide bond between two monomers,^79^ and DTT can be used to reduce the di-sulfide bond and turn a dimer into two monomers.^53,80^ It is worth mentioning again that various oligomers may form at the silicone oil interface. According to the hypothesis we proposed, here we will only consider BSA monomers and dimers. To further test our SFG and Hamiltonian spectral analysis approach to study the BSA dimer/monomer, we subjected adsorbed BSA molecules on silicone oil to DTT. Specifically, after the above SFG experiments on adsorbed BSA on silicone oil, we replaced BSA solution in contact with silicone oil with water and replaced the water with fresh water for additional two times to remove BSA solution left on the silicone oil surface. We then brought the silicone oil (with adsorbed BSA) in contact with a 50 mM DTT solution and SFG ppp and ssp spectra were collected from the silicone oil/DTT solution interface, as shown in Fig. 4a and b (red spectra).

We also conducted a separate SFG experiment using BSA-DTT mixture solution. BSA and DTT solutions were mixed to reach a concentration of 0.1 mg mL^−1^ of BSA and 50 mM of DTT. A prism with a silicone oil film was then placed in contact with the BSA-DTT mixture and SFG ppp and ssp spectra were collected from the silicone oil/BSA-DTT mixture solution interface, as shown in Fig. 4a and b (blue spectra). For comparison purpose, SFG spectra collected from the original silicone oil/BSA solution interface are replotted in Fig. 4a and b (black spectra).

Fig. 4 shows clearly that the SFG spectra collected from the adsorbed BSA on silicone oil treated with DTT solution and those collected from the silicone oil/BSA-DTT mixture solution interface are quite similar, but are very different from the original SFG spectra collected from the silicone oil/BSA solution interface (without DTT treatment). This demonstrated that DTT can change the BSA behavior on the silicone oil surface. As we reported above, adsorbed BSA on silicone oil mainly adopts the dimer form. Since DTT can dissociate the BSA dimer, the different SFG spectra before and after DTT treatment indicate that at least some of the BSA dimers changed to monomers. Fig. 4 shows that the 1630 cm^−1^ side peak detected from the original silicone oil/BSA solution interface disappeared after the DTT treatment. Similar SFG spectra obtained from the silicone oil/pre-mixed BSA-DTT solution interface and the adsorbed BSA after the DTT treatment likely indicate that DTT has a similar effect on BSA in solution and adsorbed BSA at the interface.

We fitted SFG spectra collected from adsorbed BSA treated by DTT to remove the non-resonant contribution and reconstructed the resonant SFG spectra. We then compared the spectra to the calculated SFG spectra using the Hamiltonian method based on the BSA monomer crystal structure to generate heat map (shown in Fig. S4†). However, we found that the heat map cannot provide good matching for any of the BSA monomer orientations. More details of the heat map and matching scores can be found in the ESI.† Clearly not all the adsorbed BSA dimer molecules after the DTT treatment turned into the monomer form. It is likely that some of the adsorbed BSA dimers dissociated into monomers because of DTT, while others stayed in the dimer form, leading to a mixture of BSA dimers and monomers on the silicone oil. This is reasonable since the DTT treatment was conducted at room temperature. The mild reaction condition cannot result in a 100% dissociation yield.

**Fig. 5:**
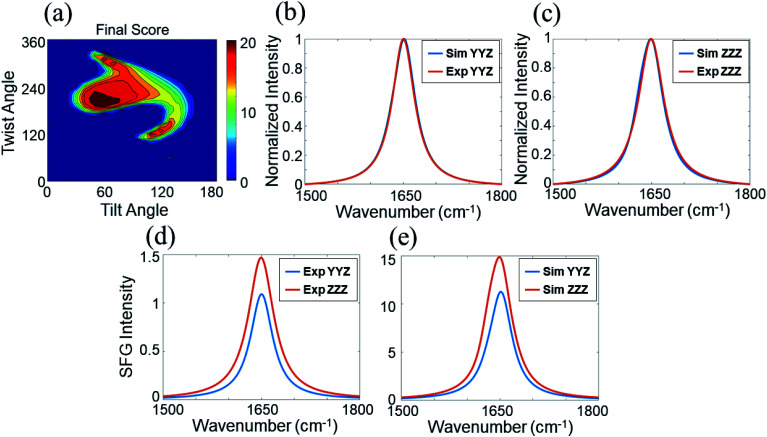
Matching qualities between the experimental data and calculated spectra based on the BSA dimer–monomer mixture of 60 : 40 ratio for BSA after the DTT treatment: (a) heat map showing the matching quality between the reconstructed resonant SFG spectra of BSA after the DTT treatment and calculated SFG spectra using the BSA dimer–monomer mixture ratio of 60 : 40. The blue spectra in (b) and (c) show the calculated SFG *yyz* (b) and *zzz* (c) spectra of BSA after the DTT treatment with the best matching quality with the reconstructed resonant SFG spectra (shown in red). The SFG spectra shown in (d) are replotted fitted experimentally collected SFG spectra. The spectra shown in (e) are calculated SFG *yyz* (blue) and *zzz* (red) spectra with the best matching quality. The matching qualities of the spectral features can be seen from (b) and (c), while the matching quality for the ssp and ppp intensity ratio can be seen from (d) and (e).

We then calculated SFG spectra using the Hamiltonian method based on a 60 : 40 mixture of the BSA dimer and monomer as a function of monomer orientation, assuming that the dimer retains the same orientation before and after the DTT treatment. The calculated SFG spectra were compared to the reconstructed SFG spectra from experimental data to generate a heat map (shown in Fig. 5a). It was found that the matching quality in the heat map for certain monomer orientation can be very high. Fig. 5 shows the comparison between the calculated and reconstructed experimental spectra with the highest matching score, indicating that the spectra match with each other very well. Assuming before the DTT treatment, the original BSA dimer surface coverage is A. If 0.25 A BSA dimers dissociate into BSA monomers after the DTT treatment, the surface coverages of the dimers and monomers will be 0.75 A and 0.50 A (One dimmer generates two monomers), which is 60 : 40. This suggests that likely 25% of the BSA dimers adsorbed on silicone oil can be dissociated by DTT to turn into BSA monomers.

**Fig. 6:**
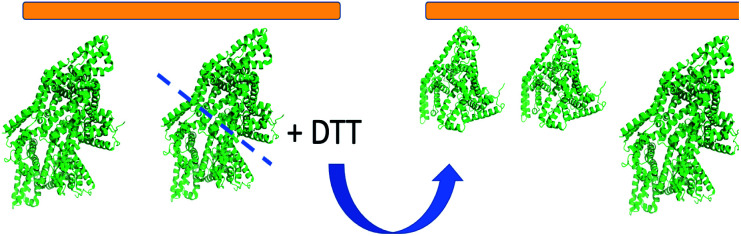
Schematic showing the interfacial reaction between DTT and the BSA dimer on the silicone oil surface and the deduced orientations of the BSA monomer and BSA dimer on the silicone oil surface after the DTT treatment.

Fig. 6 shows the schematic of some adsorbed BSA dimers on silicone oil dissociated into BSA monomers. The orientations of the BSA dimers and BSA monomers displayed in Fig. 6 are the orientations with the best matching scores in the heat maps. Both BSA dimers and monomers are tilting on the silicone oil surface, with similar orientations. The adsorption regions or contact areas for the BSA dimers and monomers on the silicone oil surface are also similar. The DTT molecules can cleave an adsorbed BSA dimer into two monomers, but do not change the adsorbed BSA orientation on silicone oil. It is worth mentioning that there is another most likely BSA monomer orientation that can be deduced from the heat map shown in Fig. 5 with an opposite absolute orientation from the one shown in Fig. 6. We believe that orientation is unlikely, which can be seen from our discussion below on the BSA–silicone oil interactions. In addition to the 60 : 40 mixture, we also compared the calculated SFG spectra with the fitted experimental spectra for other BSA dimer–monomer mixture ratios. The differences between the calculated and fitted experimental spectra of other mixtures are larger than those from the mixture with a 60 : 40 ratio (ESI, Table S1†).

It is worth mentioning that since protein aggregation may occur through various ways,^78^ DTT may reduce the disulfide bonds, and may also be considered an external modulator of the dynamic oligomerization pathway of BSA. Nevertheless, the use of DTT in this study is aimed to decrease the BSA dimer/monomer ratio, which is successful for interfacial BSA according to our SFG results presented above and also for bulk BSA according to the PAGE data shown in the ESI.†

### Circular dichroism spectroscopic study result

**Fig. 7:**
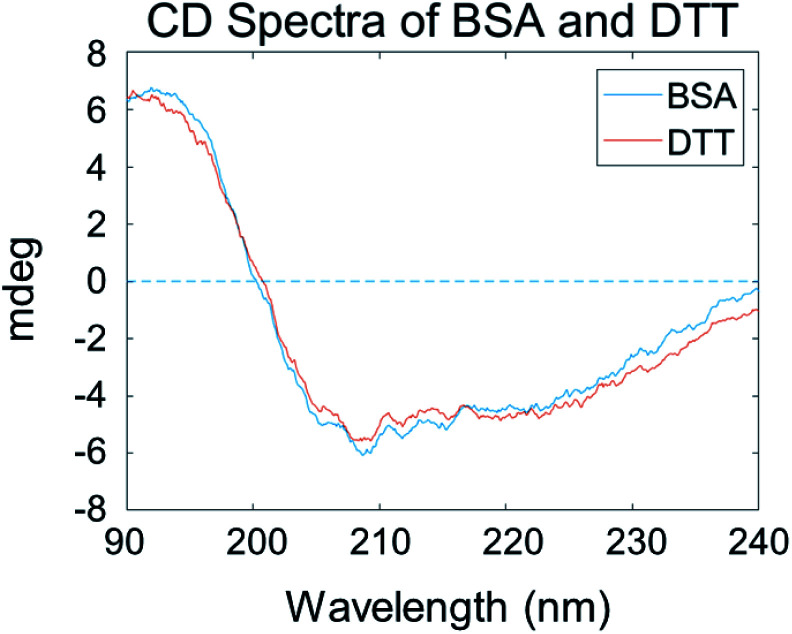
CD spectra of BSA on silicone oil before and after the DTT treatment.

The above SFG data analysis indicates that DTT can cleave BSA dimers into monomers. We believe that the DTT treatment would not substantially change the BSA molecular structure, *e.g.*, the secondary structure. To confirm this, we studied the secondary structures of adsorbed BSA on silicone oil by CD experiments. Fig. 7 shows that the CD spectra collected from adsorbed BSA on silicone oil before and after the DTT treatment are very similar, both of which exhibiting two negative peaks at 210 and 222 nm – the two characteristic peaks of the α-helical structure. Similar CD spectra from adsorbed BSA before and after the DTT treatment indicate that there is no significant change in the secondary structures of the adsorbed BSA, suggesting that DTT does not alter the BSA conformation substantially and the spectral changes observed in the SFG experiments should mainly be due to the change from a BSA dimer to monomer as analyzed above.

### Solvent accessible surface area analysis

BSA molecules have been extensively used as passivation coatings to prevent nonspecific adsorption of other molecules on the surface.^52^ We have shown that the excellent passivation properties of BSA are related to its strong surface hydration, contributed by its high surface coverage of amino acids glutamic acid (E) and lysine (K).^81^ E and K behave as zwitterionic pairs and provide anti-fouling properties.^82^ Here we quantified the E/K ratio on the surface of BSA by calculating the solvent accessible surface area (SASA) to further understand the adsorption behavior of BSA on silicone oil. The number of E and K residues in the sequence considers both E/K on the protein surface and E/K in the interior of the protein, while E/K SASA considers the effect of protein surface E/K. We propose that the E/K SASA is more significant than the number of E and K residues in the sequence since the BSA adsorption behavior is mainly affected by the outer surface of the protein.

**Fig. 8:**
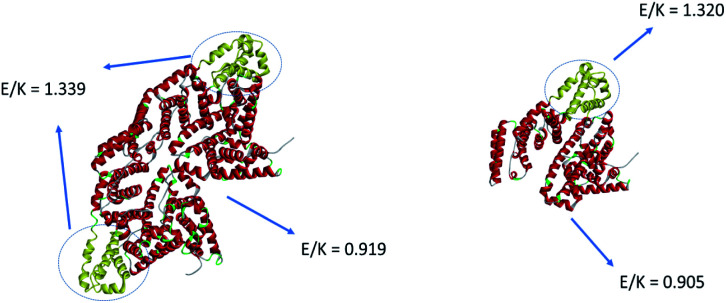
Solvent accessible surface area (SASA) analysis of the E/K ratio of the adsorption site and other parts of a BSA dimer and a BSA monomer.

E and K SASA considered the effect of surface E and K on BSA while the total number of E or K in the sequence may not be related to the BSA surface properties. The SASA calculation was performed in VMD^83^ and the result is shown in Fig. 8. The orientation of BSA shown in Fig. 8 is the same as the deduced BSA orientation displayed in Fig. 3 above. We selected the adsorption region on the BSA dimer which is adjacent to the silicone oil surface (yellow region in Fig. 8) and then calculated the ratio of SASA between E and K to be 1.339. Then all residues other than the adsorption site (red region in Fig. 8) were selected and the calculated SASA E/K ratio is 0.919. Since E and K can behave as a zwitterionic pair, the protein (or a part of the protein) will exhibit more anti-fouling properties if the E/K ratio is closer to 1. From Fig. 8, we can see that the E/K SASA ratio of the adsorption site is higher than 1 and that of other parts of BSA is closer to 1, suggesting that the adsorption site has less zwitterionic properties and thus may have a stronger interaction with the silicone oil surface. This explains why a BSA dimer tends to tilt on the silicone oil surface with the region marked in yellow in contact with the silicone oil.

Besides the E/K ratio analysis, we also measured the SASA of positively charged, negatively charged, and hydrophobic amino acid residues. The result is summarized in Table 1. It has been shown that the silicone oil carries negative charges,^84,85^ and BSA is slightly negatively charged in a pH = 7 solution since the isoelectric point of BSA is 4.8. From the SASA values of positively and negatively charged amino acids as shown in Table 1, we can see that both the BSA adsorption part and the other part of the BSA surface are negative. If charge interactions play adominating role in the protein–silicone oil interaction, then BSA should not be adsorbed to the silicone oil surface. The observation of BSA adsorption on silicone oil indicates that the charge interaction does not play a major role in the BSA adsorption phenomenon. Since the BSA adsorption part has much less amino acids compared to the rest of the BSA protein, the SASA value of the other parts of BSA is much larger than that of the adsorption part. For a better comparison, the SASA values are divided by the number of amino acids to obtain the average value for each amino acid. The average SASA result is shown in Table 2.

**Table 1:** Solvent accessible surface area (SASA) calculation results of positively charged, negatively charged, and hydrophobic amino acids in the adsorption and the other parts of the BSA dimer and monomer

SASA value (Å^2^)	Dimer adsorption part	Dimer rest part	Monomer adsorption part	Monomer rest part
Positively charged amino acid (R,K,H)	2072.63	13 407.80	1040.10	6883.96
Negatively charged amino acid (D,E)	3403.04	13 864.99	1667.00	7030.07
Hydrophobic	2216.07	11 022.60	1106.81	5503.20

**Table 2:** Average solvent accessible surface area results of positively charged, negatively charged, and hydrophobic amino acids in the adsorption and other parts of the BSA dimer and monomer

SASA value (Å^2^)	Dimer adsorption part	Dimer rest part	Monomer adsorption part	Monomer rest part
Positively charged amino acid (R,K,H)	23.82	27.03	11.96	13.88
Negatively charged amino acid (D,E)	39.11	27.95	19.16	14.17
Hydrophobic	12.74	11.11	12.72	11.10

Table 2 shows that the adsorption parts of both the BSA dimer and monomer have higher average negative SASA values compared to the average positive SASA values in these parts, as well as the average negative SASA values of the other BSA parts, again suggesting that the negative charge repulsion between the silicone oil surface and BSA is not the determining factor in BSA adsorption onto silicone oil. The average SASA values of the hydrophobic amino acids of the adsorption part are slightly higher than those of the other BSA parts for both the dimer and the monomer, which indicates that the hydrophobic interaction between BSA and the silicone oil is also important. To summarize, we believe that the E/K ratio or the zwitterionic effect and the hydrophobic interaction are the two main factors that affect the adsorption and the orientation of BSA on the silicone oil surface.

## Conclusions

It is important to understand protein behavior at interfaces. Protein aggregation at interfaces may greatly reduce protein function. To the best of our knowledge, the methodology to study protein aggregation at buried interfaces *in situ*, *e.g.*, identify protein monomers or dimers at the buried solid/liquid or liquid/liquid interfaces, is lacking. Here, using widely studied and extensively applied BSA molecules, we demonstrated that SFG study along with Hamiltonian data analysis can be used to distinguish the protein monomer, dimer, and monomer–dimer mixture at interfaces *in situ* in real time. It is worth reiterating that protein–surface interactions can be complicated, leading to the formation of oligomers beyond dimers. Here we hypothesized that the dimer is the most probable oligomer form, and tested this hypothesis using SFG data analysis based on the monomer and dimer structures. As shown above, our data analysis could well interpret the SFG data. However, we could not completely rule out the formation of other BSA oligomers on silicone oil, which is beyond the scope of this study.

Our studies reported here demonstrated that adsorbed BSA molecules on silicone oil are mainly in the dimer form. After the DTT treatment, about 40% of the BSA dimers turn into monomers because DTT can cleave the disulfide bond, which connects the two monomers, in the BSA dimer. The CD spectra collected from the adsorbed BSA on silicone oil show similar secondary structures of BSA on silicone oil before and after the DTT treatment, indicating that the DTT treatment does not alter the adsorbed BSA conformation, well correlated with our explanations on the SFG spectral change of adsorbed BSA on silicone oil after the DTT treatment.

It is worth mentioning that here we used the BSA crystal structure in the Hamiltonian calculations for SFG data analysis. Since BSA is a globular protein with a rigid structure, we believe that the use of the BSA crystal structure in the calculation is reasonable, which can be proved by the excellent matching results between the experimental data and the calculated results using the Hamiltonian approach based on the BSA crystal structure. For “soft proteins” which may undergo substantial conformational changes at interfaces, the use of the protein crystal structure for Hamiltonian calculations may lead to large errors. Under such situations, it is necessary to use the interfacial structure of the protein, *e.g.*, obtained from molecular dynamics simulation, to calculate SFG spectra with the Hamiltonian method. The combined computer simulation and SFG studies can be used to identify the most likely protein conformation and orientation, as we demonstrated previously.^60^ Such a combined study can also verify the conclusions obtained from the simulation, and provide more detailed and more reliable structural information from SFG studies. Although this research only shows the capability of SFG to study BSA monomers and dimers, we believe that SFG is able to examine protein aggregates with more complicated structures such as trimer, tetramers *etc.* with the help of computer simulations.

A substantial amount of research has been performed to apply various bulk analytical and biochemical methods to study complicated protein aggregates in bulk solutions.^86,87^ It is possible to study such protein aggregates at interfaces using the SFG and Hamiltonian approach if reasonable models can be used for Hamiltonian calculations through MD simulations or other methods, which is beyond the scope of the current investigation.

The developed methodology in this research is generally applicable to study the behavior of other proteins such as antibody protein drug molecules on silicone oil, which will lead to a great impact on pharmaceutical research and applications. The developed method can also be applied to study protein molecules on other surfaces and interfaces beyond the silicone oil surface, and will help develop further understanding of interfacial protein behavior and its relationship with biocompatibility of biomaterials, performance of antifouling coatings, protein fouling on membranes used for bio-separation, the sensitivity of biosensors, *etc.*

## Materials and methods

### Samples and materials

BSA and dithiothreitol (DTT) samples were purchased from Sigma-Aldrich (St. Louis, MO). The BSA solution concentration used in this study is 0.1 mg mL^−1^ in deionized water. DTT solution with a concentration of 50 mM was prepared by dissolving DTT in deionized water. Medical-grade silicone oil (360 Medical Fluid 1000 Cst) was purchased from Dow Corning (Midland, MI). CaF_2_ prisms used for the SFG study were bought from Altos Photonics (Bozeman, MT).

### Silica-coated CaF_2_ prisms

Silica coated CaF_2_ prisms were used as substrates to prepare silicone oil films. For cleaning, CaF_2_ prisms were soaked in toluene for 24 h, immersed in acetone and methanol each for 10 min, rinsed thoroughly with deionized water (18 MΩ cm), and dried under a nitrogen gas flow. Then the prisms were treated with oxygen and argon plasma (Glen 1000P Plasma Cleaner) to remove (if any) residue organic contaminants. Next, the cleaned prisms were transferred into the chamber of an Angstrom Engineering Evovac Evaporator to deposit 100 nm of SiO_2_ film on each CaF_2_ prism *via* physical vapor deposition.

### Preparation of a silicone oil thin film

A silicone oil thin film was prepared by spin-coating silicone oil onto the silica-coated CaF_2_ prism by a previously published method.^65^ Silicone oil was dissolved in toluene to make a concentration of 0.5 wt% solution. The silicone oil solution was dropped on the prism surface and spin-coated at 2000 rpm for 30 s. The silicone oil coated prism was dried in air. The silicone oil thickness was measured (EP^3^ Nanofilm, Germany) to be about 25 nm by using ellipsometry.

### Circular dichroism

The secondary structure of adsorbed BSA on silicone oil was studied by using CD spectra collected with a J-1500 CD spectrometer (JASCO Inc., Japan). For CD studies, a silicone oil film was prepared on a quartz slide by the same spin-coating procedure used to prepare the silicone oil film on the prism for SFG studies presented above. To prepare adsorbed BSA on silicone oil samples for the CD study, BSA solution was dropped on the silicone oil surface on quartz slides and then washed with deionized water for 30 min to remove loosely adsorbed BSA. Seven quartz slides with the silicone oil film and with adsorbed BSA were stacked for measuring CD spectra to improve the signal/noise ratio. After the CD data collection was completed, 50 mM DTT solution was dropped on the silicone oil surface and the CD spectra of adsorbed BSA after DTT treatment were collected. Each CD spectrum was collected from 190 to 240 nm with 1 nm step. The scan rate was set at 50 nm min^−1^ and each presented CD spectrum was obtained by averaging 10 scans.

## Data availability

All data needed to evaluate the conclusions in the paper (SFG and CD spectra, PAGE results) are present in the paper and/or the ESI. Additional data related to this paper may be requested from the author zhanc@umich.edu on reasonable request

## Author contributions

T. L. and Z. C. conceived the project and T. L. designed the SFG experiments; T. L. performed the SFG experiments. W. G. contributed to the SFG experiments. E. N. G. M., P. M. D. and Y. X. designed the native-PAGE experiments. P. M D. performed the native-PAGE experiments. T. L. and Y. X. performed the data analysis of native-PAGE; T. L. and Z. C. principally wrote the manuscript with input from all.

## Conflicts of interest

There are no conflicts to declare.

## Supplementary Material

SC-013-D1SC04300E-s001
