# Supplementary material for: Probing protein aggregation at buried interfaces: distinguishing between adsorbed protein monomers, dimers, and a monomer–dimer mixture in situ
Source: Chem Sci. 2021 Dec 21;13(4):975–84. doi: 10.1039/d1sc04300e (PMC8790787; doi:10.1039/d1sc04300e)
Supplement: SC-013-D1SC04300E-s001 [file SC-013-D1SC04300E-s001.pdf]

Electronic Supplementary Material (ESI) for Chemical Science.
This journal is © The Royal Society of Chemistry 2021

Supporting Information

Probing Protein Aggregation at Buried Interfaces: Distinguishing Adsorbed Protein Monomer, Dimer, and Monomer-Dimer Mixture in Situ

Tieyi Lu, Wen Guo, Prathamesh M. Datar, Yue Xin, E. Neil G. Marsh, and Zhan Chen*

Department of Chemistry, University of Michigan, 930 North University Avenue, Ann Arbor, MI 48103, USA

*Corresponding Author: Zhan Chen, Email: zhanc@umich.edu

S1 SFG sample geometry and SFG spectra reproducibility

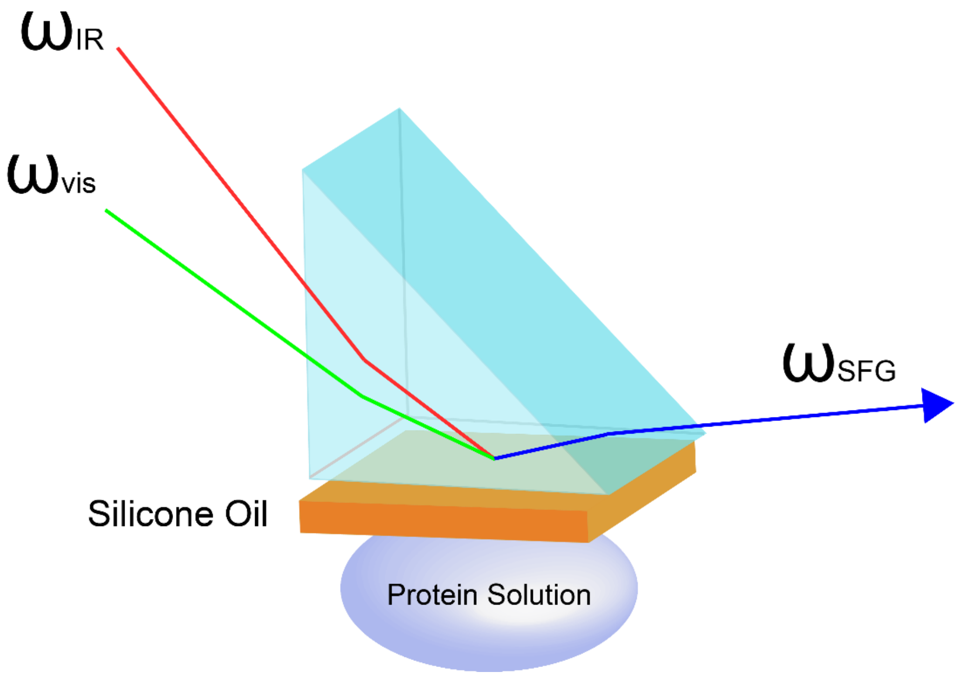

Fig. S1 Schematic of the SFG sample geometry used in this experiment.

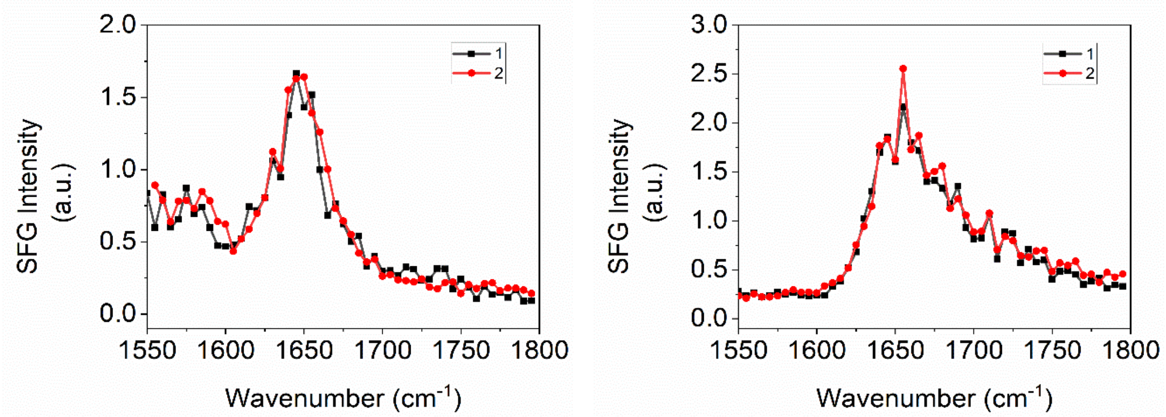

Fig. S2 SFG ssp spectra from (left) the silicone oil/BSA solution interfaces and (right) the silicone oil/BSA solution interface after the DTT treatment. The collected SFG spectra are very reproducible.

S2 Hamiltonian analysis and matching method

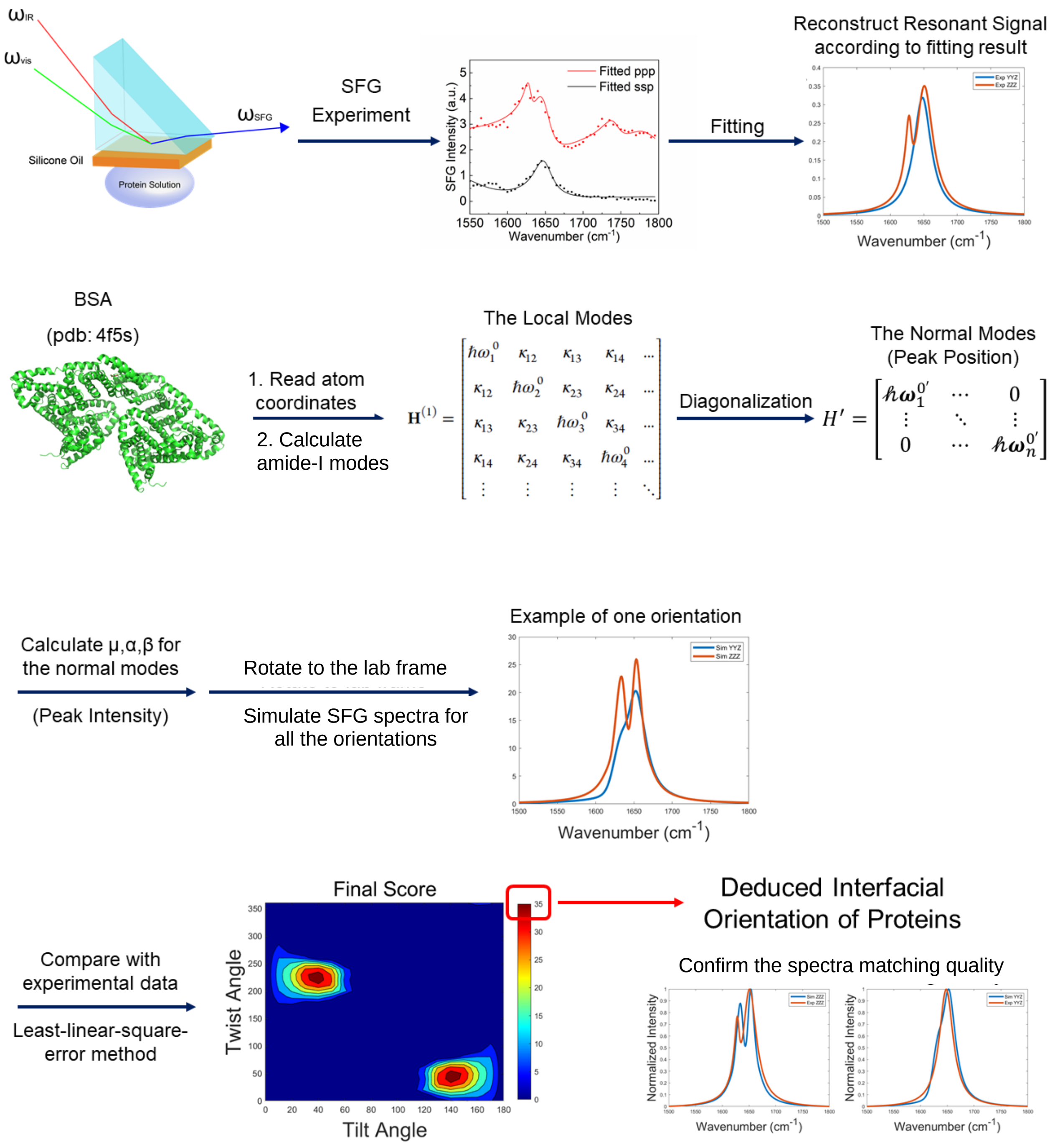

Fig. S3 Flow chart showing the Hamiltonian data analysis method used to deduce protein orientation from experimentally collected SFG spectra. μ, α, β are the IR transition dipole, Raman polarizability and SFG hyperpolarizability respectively.

The details of using the Hamiltonian method to generate SFG spectra can be found elsewhere and will not be repeated.[1] After the SFG spectra were generated as a function of protein orientation by using the Hamiltonian method, a new method (different from the published method[1]) was used to match the calculated spectra and the fitted experimentally collected spectra. Here a linear least square method was used for evaluating the matching quality between the calculated spectra and the experimental data for each protein orientation. The matching process involves several steps:

1. Fit the experimentally collected SFG spectra and reconstruct the resonant SFG spectra from the fitting parameters.
2. For spectral feature comparison for a particular protein orientation: Normalize the calculated spectra and reconstructed experimental spectra according to the highest peak intensity (at 1645 cm^{-1} for this study).
3. Calculate the square of the difference between the normalized calculated and experimental spectra at each data point, then sum all the squares for all the data points:

$$SE_1 = \sum_x \left(Y_{x,cal} - Y_{x,exp}\right)^2$$

where Y is the normalized SFG intensity, and x is wavenumber.

4. Repeat step 3 for all the orientations. Heat maps can be generated from SE_1 as a function of protein orientation for SFG ssp and ppp spectra (These maps display the matching qualities of spectral features of ssp and ppp spectra).

5. Calculate the ssp and ppp peak intensity ratio square at 1645 cm^{-1} for the calculated and measured SFG spectra as a function of protein orientation to generate a heat map (This heat map addresses the matching quality of the ssp and ppp intensity ratio):

$$SE_2 = \left(\left| \frac{Y_{1645,cal,yyz}}{Y_{1645,cal,zzz}} \right| - \left| \frac{Y_{1645,exp,yyz}}{Y_{1645,exp,zzz}} \right| \right)^2$$

6. The three heat maps can be combined to generate the overall square difference heat map.

$$SE_{Final} = SE_{1,ssp} + SE_{1,ppp} + SE_2$$

7. Convert the square difference heat map to final score heat map. The final score of each orientation is equal to the highest square error value in the heat map subtracted by the SE_{Final} at each orientation.
8. Identify the orientation that has the highest final score (lowest square difference).

S3 Matching the fitted experimentally collected spectra of BSA after DTT treatment with calculated spectra based on the BSA monomer structure

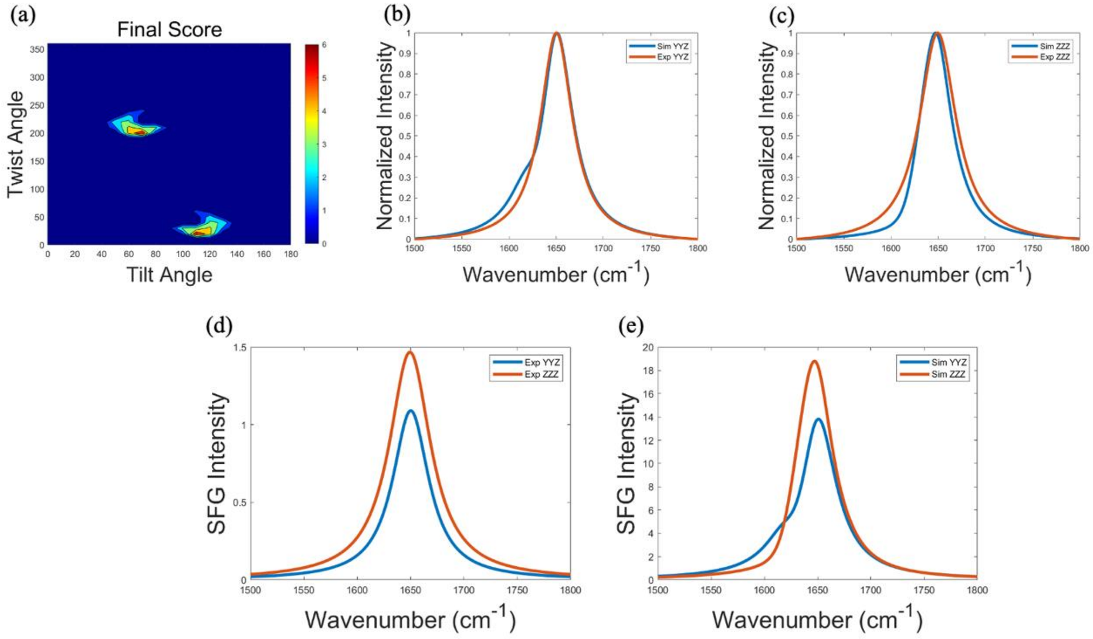

Fig. S4 Matching qualities between the experimental data and calculated spectra based on BSA monomer structure for BSA after the DTT treatment: (a) Heat map showing the matching quality between the reconstructed resonant SFG spectra of BSA after the DTT treatment and calculated SFG spectra using the BSA monomer structure. The blue spectra in (b) and (c) show the calculated SFG yyz (b) and zzz (c) spectra of BSA after the DTT treatment with the best matching quality with the reconstructed resonant SFG spectra (shown in red). The matching qualities of the spectra are much worse than those shown in Figure 5 in the main text based on BSA dimer-monomer mixture. The SFG spectra shown in (d) are replotted fitted experimentally collected SFG spectra. The spectra shown in (e) are calculated SFG yyz (blue) and zzz (red) spectra with the best matching quality. The matching qualities of the spectral features can be seen from (b) and (c), while the matching quality for the ssp and ppp intensity ratio can be seen from (d) and (e). The ssp and ppp spectra can be converted to yyz and zzz spectra after considering the Fresnel coefficients.

S4 Matching the fitted experimentally collected spectra of BSA after DTT treatment with calculated spectra based on the BSA dimer structure

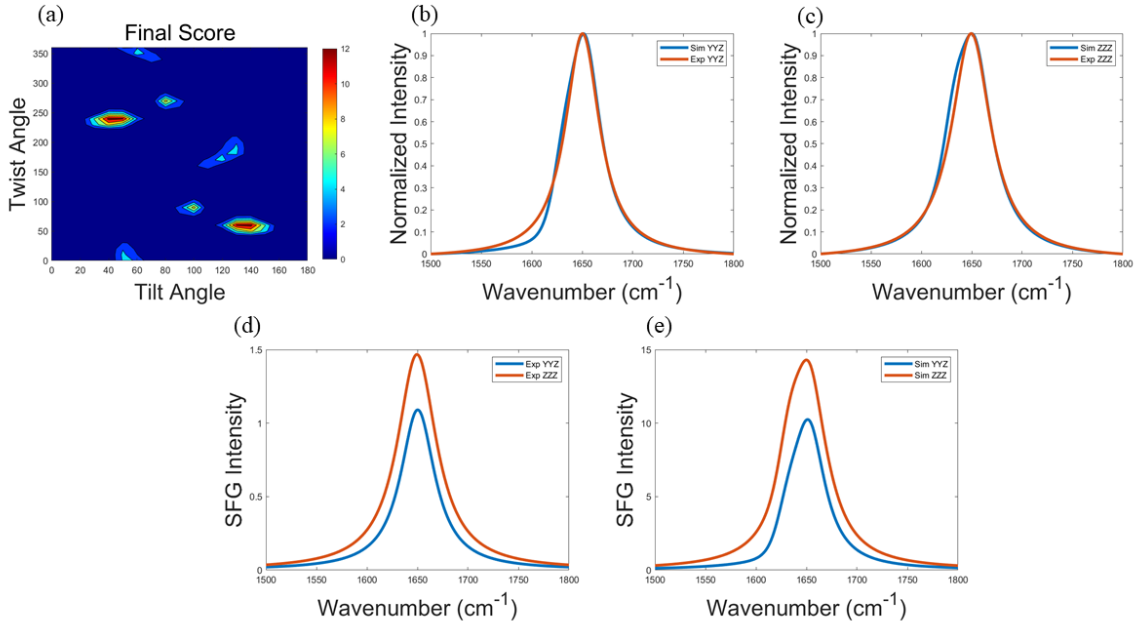

Fig. S5 Matching qualities between the experimental data and calculated spectra based on BSA dimer structure for BSA after the DTT treatment: (a) Heat map showing the matching quality between the reconstructed resonant SFG spectra of BSA after the DTT treatment and calculated SFG spectra using the BSA dimer structure. The blue spectra in (b) and (c) show the calculated SFG yyz (b) and zzz (c) spectra of BSA after the DTT treatment with the best matching quality with the reconstructed resonant SFG spectra (shown in red). The matching qualities of the spectra are much worse than those shown in Figure 5 in the main text based on BSA dimer-monomer mixture. The SFG spectra shown in (d) are replotted fitted experimentally collected SFG spectra. The spectra shown in (e) are calculated SFG yyz (blue) and zzz (red) spectra with the best matching quality. The matching qualities of the spectral features can be seen from (b) and (c), while the matching quality for the ssp and ppp intensity ratio can be seen from (d) and (e).

To quantify which BSA dimer-monomer mixture ratio can generate calculated SFG spectra with the best matching quality with the experimental data, we varied the dimer-monomer mixture ratio from 1:9 to 9:1, calculated SFG spectra as a function of orientation, and matched the calculated spectra with the experimental data. Table S1 lists the difference square sum of the SFG spectra with best matching quality for each dimer-monomer ratio, showing that the calculated SFG spectra based on the BSA dimer-monomer ratio of 60:40 has the lowest value of the square difference (best matching quality) among all the ratios.

This provides evidence that the silicone oil surface is likely covered by 60% BSA dimer and 40% monomer after the DTT treatment.

Dimer:monomer ratio	1:9	2:8	3:7	4:6	5:5	6:4	7:3	8:2	9:1
Sum of the square of the difference	2.048	1.578	1.204	0.7161	0.3888	**0.3142**	0.4559	0.7809	1.263

Table S1. The sum of the difference square of the SFG spectra with best matching quality for each dimer-monomer ratio. The calculated SFG spectra based on the BSA dimer-monomer ratio of 60:40 has the lowest value of the square difference (or the best matching quality).

S5 SFG spectral fitting parameters

	ssp	ppp
Offset	0.16	-0.07
NR	-0.378	-0.643
A1	16.7	2.26
x01	1648	1629
w1	15.8	5.4
A2	0.454	16.5
x02	2195	1650
w2	50.0	15.7
A3	31.5	21.2
x03	1546	1786
w3	50.0	30.7
A4	0.520	6.43
x04	1501	1742
w4	0.323	14.0

Table S2. Fitting parameters of the SFG spectra of BSA on silicone oil shown in Figure 1(b).

	BSA / DTT		BSA-DTT Mix	
	ssp	ppp	ssp	ppp
Offset	-0.48	-0.43	-0.043	-0.099
NR	0.432	0.258	0.327	0.195
A1	41.4	57.2	31.7	40.2
x01	1643	1647	1646	1650
w1	25.5	27.1	26.9	27.9
A2	25.1	17.5	19.8	13.1
x02	1577	1577	1531	1552
w2	50.0	42.2	50.0	50.0
A3	34.5	26.8		
x03	1504	1511		
w3	50.0	50.0		

Table S3. Fitting parameters of the SFG spectra of BSA on silicone oil after the DTT treatment and the BSA-DTT mixture shown in Figure 4.

S6 PAGE experiments

We performed native PAGE experiments to determine BSA monomer and dimer amounts in the BSA solution before and after silicone oil surface contact, and before and after the DTT treatment. A BSA solution of concentration 1.0 mg/mL with a volume of 10 mL was prepared in a glass container. A silica window coated with silicone oil was placed in the above BSA solution for 30 minutes. The silicone oil surface was then removed from the BSA solution. 10 μL of the BSA solutions before and after the silicone oil contact was used in the native PAGE experiment. A new BSA solution of concentration 1.0 mg/mL with a volume of 10 mL was prepared in a glass container. 77 mg of DTT was added to the BSA solution. A silica window coated with silicone oil was placed in the above DTT added BSA solution for 30 minutes. The silicone oil surface was then removed from the DTT added BSA solution. 10 μL of the DTT added BSA solutions before and after the silicone oil contact each was used in the native PAGE experiment.

The native PAGE experiment was performed using a 4-20% Mini-PROTEAN TGX polyacrylamide gel (Bio-Rad). 10 μL of samples were mixed with 20 μL Native sample buffer (Bio-Rad) and 10 μL of this was loaded onto gels. Electrophoresis was performed at 30 V and 4℃ for 14 hrs in Tris/Glycine buffer (Bio-Rad). The gel was stained using InstantBlue Commassie Protein Stain (Novus Biologicals) and visualized via ChemiDoc Touch Imager (Bio-Rad). Protein bands were quantified using Image Lab (Bio-Rad).

We performed several PAGE experiments and qualitative reproducible results were obtained. Figure S4 shows the results from one example run. The four samples are BSA solutions before (1) and after (2) silicone oil exposure and BSA solutions with DTT added before (3) and after (4) silicone oil exposure. Table S4 presented the quantitative results obtained from the PAGE data shown in Figure S4. Figure S4 clearly shows that all the samples are dominated by the BSA monomers, with substantial amounts of BSA dimers. After the DTT treatment, the dimer amounts in the solution reduced noticeably. However, for the BSA solution before and after silicone oil surface contact, the dimer/monomer ratios are not very different. Table S4 shows representative band intensities obtained after quantifying images for stained gels.

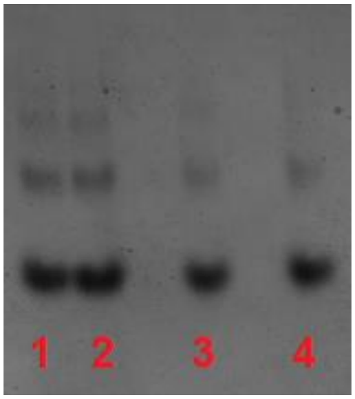

Figure S6 Native PAGE results of BSA solution before (1) and after (2) silicone oil surface contact, and DTT treated BSA solution before (3) and after (4) silicone oil surface contact

	(1) BSA	(2) BSA Oil	(3) BSA DTT	(4) BSA Oil DTT
**Dimer**	4.6×10^6	5.0×10^6	2.5×10^6	2.5×10^6
**Monomer**	1.9×10^7	2.3×10^7	1.7×10^7	1.9×10^7
**D/M**	1:4.1	1:4.6	1:6.8	1:7.6

Table S4 Quantitative analysis results of the PAGE experiments. The first two rows are intensities of the BSA monomer and dimer bands in the gel. The third row is the ratios of the intensities from dimer vs. monomer.

Table 4 shows that the dimer/monomer ratios decreased after the DTT addition, indicating that DTT reduced some dimer molecules to monomers, as expected. We do not think that the native-PAGE experiments can provide quantitative correlations to SFG data. For quantitative correlations, after the silicone oil exposure, protein molecules (total intensity) should decrease, which was not observed. In the literature, similar methods were used to determine the possible monomer and dimer amounts adsorbed onto a surface. That is, a bulk method (e.g., PAGE) was used to measure the BSA dimer and monomer concentrations or amounts in a BSA solution. Then a surface was placed into contact with the solution. After that, the bulk solution (after the surface contact) was analyzed again using the bulk method to determine the BSA monomer and dimer amounts. According to the difference before and after the surface contact, possible amounts of BSA monomer and dimer on the surface were deduced. Unfortunately the results obtained from this method may not be related to the amounts of BSA monomer and dimer on the (silicone oil) surface because BSA monomer and dimer can be re-equilibrated in the solution after the

surface contact. Also, the interfacial interactions between adsorbed BSA and the surface may lead to dimer dissociation or monomer dimerization on the surface, which cannot be probed using the PAGE method.

S7 Reference orientations of the BSA dimer and BSA monomer

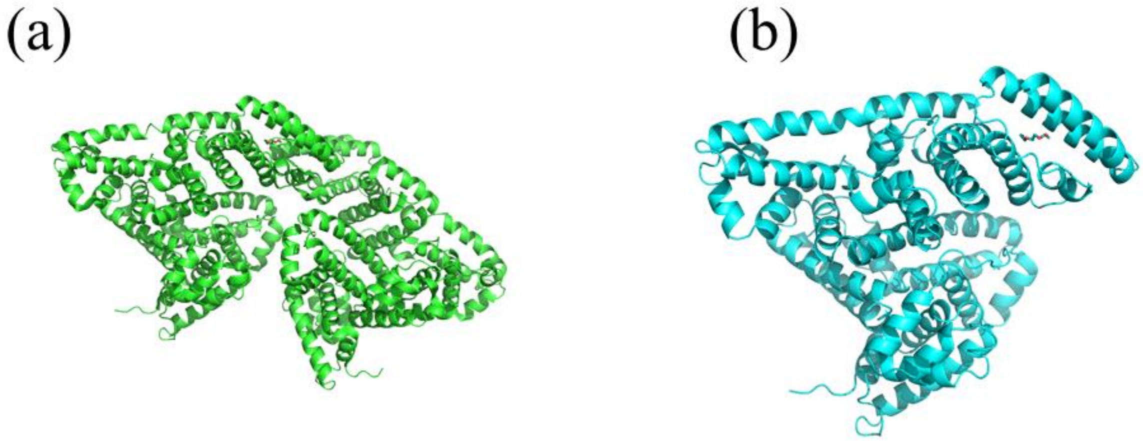

Fig. S7 Reference orientations (Tilt=0, Twist=0) of BSA (a) dimer and (b) BSA monomer.

Reference

1. W. Guo, X. Zou, H. Jiang, K. J. Koebke, M. Hoarau, R. Crisci, T. Lu, T. Wei, E. N. G. Marsh and Z. Chen, *The Journal of Physical Chemistry B*, 2021. 125, 28, 7706–7716
